# Household food insecurity and coping strategies among pensioners in Jimma Town, South West Ethiopia

**DOI:** 10.1186/s12889-018-6291-y

**Published:** 2018-12-14

**Authors:** Misgana Asesefa Kisi, Dessalegn Tamiru, Melese Sinaga Teshome, Meseret Tamiru, Garumma Tolu Feyissa

**Affiliations:** 1 Department of Population and Family Health, Jimma University, Jimma, Ethiopia; 2 Department of Health, Behaviour and Society, Jimma University, Jimma, Ethiopia

**Keywords:** Pensioners, Food insecurity, Jimma, Ethiopia, Africa

## Abstract

**Background:**

Ethiopia is currently facing new challenges related to food insecurity among the urban poor. Pensioners are segments of the population with reduced income and working capacity because of advancement of age and other related problems. There is no empirical evidence on Jimma Town pensioner’s household food insecurity and coping strategies.

**Methods:**

A cross-sectional study was conducted among households in Jimma Town living on an income obtained from a pension from March 01–28, 2017. Data were collected from 399 randomly selected participants. Data were entered into EPi-Data version 3.1 and analyzed using SPSS Version 20.0. Variables with *p* ≤ 0.25 in the bivariate analyses were entered into a multivariable regression model to control for confounding variables.

**Results:**

Nearly, 83.5% of households were food insecure. The odds of food insecurity among households with heads attending secondary school and above was 78% lower when compared to that of households with uneducated household heads (AOR = 0.22, 95% CI: 0.97 to 0.49). The odds of food insecurity among households headed by merchants was 91% lower when compared to that of households headed by guards (AOR = 0.09, 95% CI: 0.03, 0.29). Food insecure households were using coping strategies such as changing consumption patterns (44%), eating inexpensive foods (72.4%), reducing meal frequency (62.4%) and selling household assets, such as household food utensils (30.8%). The odds of food insecurity among households having large family size (≥ 7) was 3.74 times higher when compared to that of households with family size less than three (AOR = 3.74(1.27_,_ 10.99).

**Conclusions:**

Household food insecurity was associated with having households headed by uneducated, widowed and guard household heads and having large family size. Food insecure households used both consumption and asset-based coping strategies such as eating less preferred, lower quality or less expensive foods and receiving donation from relatives or friends. Government policies should consider revising the current social protection scheme for pensioners. Special attention should be given to widow pensioners and pensioners with low educational status and with large family sizes.

## Background

According to the United Nations (UN), a service of 60 years is required to qualify as a pensioner. This corresponds to Ethiopia’s official retirement age [1]. Pension is a remittance coming from savings accrued during the working life. Ethiopia is undergoing a demographic transition and population above the age of 60 years is estimated to raise to 6.8% by 2050 [1, 2]. Currently, older people including pensioners are forced to be food insecure, unless they have adequate family and community support. Part of the reason for this has to do with the gradual erosion of the social nexus and the tradition of inter-generational support due to urbanization and modernization [1–3]. As a result, food insecurity is expected to be a common encounter among this segment of the population.

Food insecurity is a situation where people experience limited or uncertain physical, social and economic access to safe, enough and nutritious food to meet their dietary desires or food preferences for healthy and active life [2]. Although there is sufficient food production in the world, there is a problem of access to it making close to 800 million chronically hungry [4, 5].

In Sub-Saharan Africa, poor access to food emanates largely from a web of factors including poverty, livelihood losses, escalation of food prices, political instability and policy gaps [5, 6]. Moreover, low input, low output and rainfed traditional production system accentuated by erratic weather conditions leading to droughts, pests and animal diseases, population pressure, weak institutional capacity, and underdeveloped infrastructure are salient drivers of chronic food insecurity in Ethiopia [6, 7].

Modest predictions show that around half of the population is expected to live in cities and urban areas including an impromptu township by 2020 [4]. Food and nutrition insecurity have been on the rise in low income countries of Sub-Saharan-Africa owing to rapid urbanization. Urban net food buyer population has been challenged by the rapid increment of food price when compared to rural populations [5, 8]. Food insecurity may lead to serious social, psychological and behavioral repercussions such that at an individual level it could manifest with feelings of alienation, powerlessness, stress and anxiety potentially leading to not only reduced productivity, but also reduced work and school performance, and reduced income earnings [9]. Households and communities who encountered acute food shortages are obliged to adopt coping strategies to meet the immediate food requirements of their families. These extreme responses may have adverse long-term impacts on households’ ability to have sustainable access to food as well as the environment [9, 10].

People in older age are particularly vulnerable due to poverty, receding health, rising health costs, insufficient access to social services, and problems of neglect [10–12]. The high prevalence of poverty among older people is related to limited ability of poor families to care for their older relatives [11]. According to the report of food security strategy of Ethiopia, urban low income, households employed in the informal sector, those outside the labor market, such as elderly, disabled, sick, some female-headed households, street children and urban poor are vulnerable to economic shocks, especially those causing food price rises [12].

Social pensions have recently been recognized as a key component of the United Nation’s Social Protection Floor (UNSPF) as an initiative to reduce global economic and financial crisis that leads to food insecurity [9]. However, as reported by different studies, pension users are elder people who are greater than 60 years and are nonproductive force of social segment due to various reasons [6, 9]. To address the food insecurity problem, the government of Ethiopia is making significant investments particularly through its productive safety net program and social protection policies [13]. Nevertheless, whether the current arrangement and social protection scheme is adequate in maintaining the food security of pensioners is not clear. Although studies have examined household food insecurity and associated factors worldwide, most focused on the general population rather than focusing specific vulnerable groups such as pension user households [9, 10]. Getting adequate information about the magnitude of food insecurity within specific segments of a population such as pensioners is vital for planning social protection schemes for this specific group of population. Therefore, this study was designed to assess the prevalence of household food insecurity and associated factors among pensioners in Jimma Town, Southwest Ethiopia.

## Methods

### Study setting and sampling

A community based cross sectional design was employed in Jimma Town, Southwestern Ethiopia from March 01–28, 2017 among pension user households. Jimma Town is located at 352 km to the southwest of Addis Ababa. According to the Central Statistical Agency (CSA, 2007) report, this town has a total population of 120,960, of whom 60,824 are males and 60,136 are females. A total of 32,191 households were counted in this town. Out of this, 6481 households are pension users. In Jimma town, 15.35% of the land was used for agricultural purpose, while 84.65% of the land in the town was used for different purposes. About 15% of Jimma town population is currently practicing urban agriculture [10, 14].

The sample size was calculated by assuming 50% of households were food insecure with 95% confidence level and 0.05 margin of error, which yielded an initial sample of 384. Since the source population was less than 10,000, we applied a population correction formula, which resulted in 363. Adding a 10% non-response rate, the final sample size calculated was 399.

After getting ethical approval, study participants were selected in the following procedure: first, we went to Ministry of Labor and Social Affairs (Southwest branch) to get permission. Following that, we went to the three stations from where pensioners collect their payment every month (United bank, Commercial nominee and Posta office) to get permission and further information about pensioners. Then, using payroll of the pensioners as sampling frame, we selected the households randomly by lottery method. Finally, we listed the study participants’ names with their kebele, house numbers/phone numbers and used local guides with data collectors to reach each household. Study participants who were unable to communicate were excluded from the study.

### Data collection procedure

Data were collected through interviewer-based structured questionnaire. The questionnaire for interview was developed first in English and then translated into Afaan Oromo and Amharic and back translated to English with independent translators to check the consistency of the questionnaires. The questionnaire consisted of socio-demographic variables, socio-economic variables and items related to food security. Respondents were interviewed at their homes by trained data collectors. Data collectors and supervisors were trained on data collection tools and how to obtain consent from clients for two days by the principal investigator. All completed questionnaires were examined every day for completeness and consistency by the principal investigator and supervisors.

### Measurements

Data on socio-economic status were gathered using items developed by the Ethiopian Demographic and Health Survey (EDHS) [15]. FANTA 2013 household food insecurity accesses scale score was used to measure the degree of food insecurity in the households in the past four weeks (30 days) [16].

### Data processing and analyses

Data were entered into EPI-data version 3.1 and exported into SPSS version 20 for analysis. The wealth index was constructed using the Principal Component Analysis (PCA) from household asset data that were gathered using items designed by the EDHS to measure wealth status [15].

The following steps were undertaken to calculate food insecurity. First, a household food insecurity accesses score was calculated for each household by summing the frequency of occurrence of the nine-food insecurity-related conditions. The nine items measure household food insecurity during the past four weeks. Each of the nine items was coded as 0 = no occurrence, 1 = rarely, 2 = sometimes, 3 = often. Hence, the total score could range from 0 to 27.

Descriptive statistics like mean, proportions and numerical summary was used. Binary logistic regression was used to identify the association between each independent variable and the outcome variable. Variables with *p* ≤ 0.25 in the bivariate analyses were entered into a multivariable regression analysis to control for confounding variables. Multivariable logistic regression models were used to determine independent predictors of pension user household’s food insecurity. Those variables having a *p*-value < 0.05 in multivariate logistic regression were considered as significantly associated with the dependent variable.

### Operational definitions

1. *A Pensioner (retiree)* is a military, or other a public servant or a private retiree who collects money monthly from commercial nominee, Post Office and united bank of Jimma Town.
2. *A mildly food insecure household* is a household that worries about not having enough food sometimes or often, and/or is unable to eat preferred foods, but do not run out of food, do not go to bed hungry, or do not go the whole day and night without eating.
3. *A moderately food insecure household* is a household that sacrifices quality of food taken more frequently, by eating a monotonous diet or undesirable foods sometimes or often, and/or has started to cut back on quantity by reducing the size of meals or the number of meals, rarely or sometimes, but it does not experience any of the three most severe conditions (running out of food, going to bed hungry, or going a whole day and night without eating).
4. *A severely food insecure household* is a household that often cuts back on meal size or number of meals, and/or experiences any of the three most severe conditions (running out of food, going to bed hungry, or going a whole day and night without eating).

## Results

A total of 399 households participated in the study with a response rate of 100%. Most (82.2%) of the households were male headed households. One-third of household heads (32.8%) were 65 years and above. The mean and median age of household heads were 57.4 and 60 years respectively. More than two-third, (67.7%) of the household heads were married (Table 1).

**Table 1:** Socio-demographic and economic characteristics of pension users in Jimma Town, South West Ethiopia, 2017

Variables	Categories	Frequency	Percent
Household Head (*n* = 399)	Head	352	82.2
	Spouse	45	11.3
	Relative	2	0.6
Age Category	≤ 44	69	17.3
	45–64	199	49.9
	≥ 65	131	32.8
Sex	Male	189	47.4
	Female	210	52.6
Religion	Orthodox	191	47.9
	Muslim	138	34.6
	Protestant	66	16.5
	Wakefata	4	1.0
Occupational status	Guard	164	41.1
	Government employee	61	15.5
	Merchant	38	9.5
	Soldier	12	3
	Housewife	124	31.1
	Others^a^	22	5.5
Educational status	Uneducated	221	55.4
	Primary school	122	30.6
	Secondary school	41	10.3
	Diploma and above	15	3.8
Marital status	Married	270	67.7
	Widowed	103	25.8
	Divorced/Separated	67	16.8
	Single/Never married	7	1.8
Household Economic Status	Poor	118	29.6
	Medium	171	42.9
	High	110	27.6

^a^Religion leader, Farmer, Not specific

**Fig. 1:**
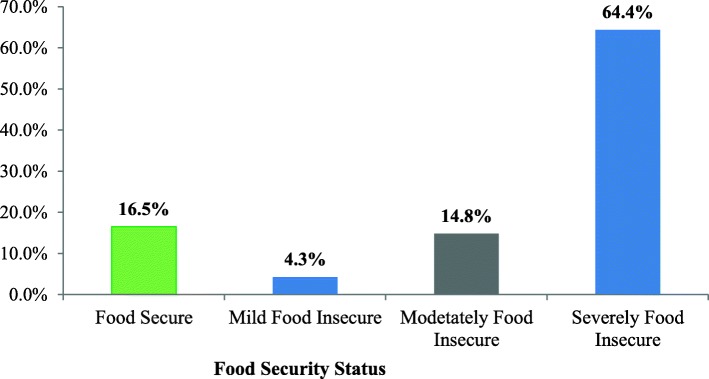
Household Food Security Status of Pension users in Jimma town, Southwest of Ethiopia, 2017

Findings of this study indicated that 66 (16.5%) of the respondents were food secure while 17 (4.3%) and 257 (64.4%) of respondents were mildly and severely food insecure respectively (Fig. 1). Of the total households, 118 (29.6%) of them reported that they have ever experienced sleeping hungry and 123 (30.8%) had no any kind of food in the house at the time of the survey. Two hundred fifty-seven (64.4%) households had just a few kinds of foods while 123 (30.8%) households reduced the amount of meal that they consumed (Table 2).

**Table 2:** Pension users’ response to Household Food Insecurity and Access Scale (HFIAS) questions in Jimma town, South West Ethiopia, 2017

HFIAS questions	Yes		No	
	Frequency	Percent	Frequency	Percent
Worry about food	333	83.5	66	16.5
Unable to eat preferred foods	303	76	96	24
Eat just a few kinds of foods	257	64.4	142	35.6
Eat foods they really do not want to eat	217	54.4	182	45.6
Reduce frequency of meal	145	36.3	254	63.7
Reduce amount of meal	123	30.8	276	69.2
No any kind of food in the house	123	30.8	276	69.2
Go to sleep hungry without eating	118	29.6	315	79
Stay a whole day and night without food	11	2.8	388	97.24

The study participants also reported that they were regulating their consumption patterns as a cost cutting measures. Among the strategies of consumption change, the most common ones were, to eat less expensive foods, 289 (72.4%) and reducing the number of meals consumed in a day, 249 (62.4%) (Table 3). Food insecure households used coping strategies, such as, borrowing money (51.9%), receiving food donation from relatives or friends (44%), selling household assets, including household food utensils (30.8%) and migration to the nearby town for wage labor (43.4%) (Table 4).

**Table 3:** Consumption Related Coping Strategies of pension users in Jimma Town, South West Ethiopia, 2017

Types of Coping Strategy	Frequency	Percentage^a^
Eat less preferred, lower quality or less expensive foods	289	72.4
Reducing consumption during each meal (pattern)	176	44
Reduce the number of meals consumed in a day	249	62.4
Selling any household assets^b^	123	30.8
Increase consumption of street food	23	5.8

^a^Since respondents might have multiple answers, the total percentage does not add up to 100

^b^This includes all household assets such as household utensils

**Table 4:** Respondent Asset and Assistance Based Coping Strategies in Jimma Town, South West Ethiopia, 2017

Type of Coping Strategies	Frequency	Percentage
Borrowing money to buy food	207	51.9
Receiving donation from relatives or friends	176	44
Migrate household member to the nearby town for wage labor	173	43.4
Selling household assets	123	30.8
Buy food on credit basis	78	19.5
Send children to work	45	11.3
Selling wood	25	6.3
Selling household livestock	21	5.3
Selling charcoal	13	3.3
Petty trading	9	2.3
Begging	7	1.75

NB: Since respondents might have multiple answers, the total percentage does not add up to 100

In the binary logistic regression analysis age, sex, marital status, educational status, access to credit service, occupational status, asset possession, existence of family members with chronic disease, means of livelihood and family size had statistically significant association with food insecurity (Table 5).

**Table 5:** Factors associated with household food insecurity among pension users in Jimma Town, Southwest Ethiopia, 2017

Variables	Food security status		COR (95%CI) AOR(95%CI)	
	Food insecure	Food secure		
Age				
≤44	59 (85.5)	10 (14.5)	0.74(0.35,1.59)	2.40(0.87, 6.65)
45–64	162 (81.4)	37 (18.6)	1	1
≥65	113 (86.3)	18 (13.7)	1.06(0.46, 2.45)	1.04(0.47, 2.29)
Sex				
Male	167 (88.4)	22 (11.6)	1.95(1.12,3.41) *	0.53(0.24, 1.15)
Female	146 (77.2)	43 (22.8)	1	1
Education status				
Secondary School and above	27 (48.2)	29 (51.8)	0.21(0.11, 0.41) *	0.22 (0.097, 0.49)^+^
Primary school (1–8)	92 (75.4)	30 (24.6)	0.10(0.052,0.24)	0.11 (0.05,0.28)^+^
No education (Illiterate)	207 (93.7)	14 (6.3)	1	1
Marital status				
Married	223 (82.6)	47 (17.4)	1	1
Divorced	16 (84.2)	3 (15.8)	1.2 (0.31, 4.01)	1.11(0.25, 4.96)
Widowed	91(88.3)	12(11.7)	1.59(0.81, 3.15)	1.10(0.44, 2.65)
Never married	4 (57.1)	3 (42.9)	0.28(0.06, 1.29)	0.17(0.02, 1.45)
Occupational status before retirement				
Merchant	23 (60.5)	15 (39.5)	1	1
Guard	153 (93.3)	11 (6.7)	0.11(0.05, 0.27) *	9.6(3.62, 25.44)^*+*^
Government employee	43 (70.5)	18 (29.5)	0.17(0.08, 0.39)	1.83(0.71, 4.73)
Soldier	8 (66.7)	4 (33.3)	0.14(0.04, 0.55)	0.36 (0.06, 2.05)
Housewife	107 (86.3)	17(13.7)	0.45(0.21, 1.01) *	0.29(0.11, 1.75)
Household economic status				
High	95 (86.4)	15 (13.6)	0.94(0.46, 1.89)	0.12 (0.52, 2.53)
Medium	149 (87.1)	22 (12.9)	0.48(0.26, 0.88)	0.62(0.29, 1.33)
Poor	90 (76.3)	28 (23.7)	1	1
Family Size				
≤3	26 (72.2)	10 (27.8)	1	1
4–6	168 (83.6)	33 (16.4)	0.41(0.06,1.30)	1.87(0.73, 4.83)
≥7	140 (64.5)	22 (35.5)	0.80(0.24, 0.97)^*+*^	2.1(1.76, 5.63) ^+^
Access to credit				
Yes	87 (87.9)	12 (12.1)	1.56(0.79, 3.05)	0.53(0.24, 1.15)
No	247 (82.3)	53 (17.7)	1	1
Means of livelihood				
Rent part of house	129 (84.3)	24 (15.7)	0.74 (0.37, 1.33)	0.30 (0.91, 1.76)
Remittance from abroad	87 (79.1)	23 (20.9)	2.88 (0.96, 8.67)	2.34 (0.93, 7.30)
Support from relatives	110 (88.7)	14 (11.3)	0.74 (0.36, 1.54)	1.53 (0.72, 3.44)
Others	8 (66.7)	4 (33.3)	1	1
Have chronic disease				
No	217 (86.5)	34 (13.5)	1.7(0.9, 2.90)	0.16(0.84, 2.94)
Yes	117 (79.1)	31 (20.9)	1	1

*Significant at < 0.001, **Significant at < 0.001

*COR* Crude Odd Ratio, *AOR* Crude Odd Ratio

Findings of multivariable analysis showed that households headed by widows were nearly two times more likely to be food insecure (AOR = 1.83, 95% CI: 1.43, 3.49) when compared to both households headed by married pensioners. The odds of food insecurity among households with heads attending secondary school and above was 78% lower when compared to that of households with uneducated household heads (AOR = 0.22, 95% CI: 0.97 to 0.49). Similarly, the odds of food insecurity among households headed by merchants was 91% lower when compared to that of households headed by guards (AOR = 0.09, 95% CI: 0.03, 0.29). The odds of food insecurity among households having a government employee as household heads was 75% lower when compared to that of households headed by guards (AOR = 0.25, 95% CI: 0.10, 0.63). The odds of food insecurity among households having large family size (≥7) was 3.74 times higher when compared to that of households having less than three family members (AOR = 3.74(1.27_,_ 10.99) (Table 5).

## Discussion

We found that the prevalence of household food insecurity was 83.5% among pension user households in Jimma Town, which is considerably higher than the national figure (35%) reported by the Ethiopian Health and Nutrition Research Institute (EHNRI) [1, 16]. The study by EHNRI used sample from the general population. This implies that the level of food insecurity among pension users is higher than the general population. In line with this study, high levels of food insecurity had also been documented in other poor urban settings in the developing countries [17]. The higher level of food insecurity identified in our study could be because most of the respondents were old or disabled and depended on the limited pension they receive. Additionally, their food supplies depended on the market values of the foods [1]. This underscores that the current social protection scheme is not adequate in maintaining the food security of pensioners.

Households headed by uneducated household heads were more likely to be food insecure when compared to households headed by those individuals who have attended secondary school and above. Previous studies have also shown that educational status has significant input to household food security status. The possible explanation is that, it may be due to the fact that uneducated heads are less likely to be employed especially in the context of the present global economic crisis [17–19].

In the current study, having a large family size was significantly associated with household food insecurity which is consistent with other studies [20–22]. This may be mainly due to large proportion of dependent household members which is not proportional to the available resource, the high unemployment rate in the town and less opportunity of self-employing scheme developed in the area, making households relay on limited resources [19, 23, 24]. It was also observed that households headed by widows were more likely to be food insecure when compared to households headed by married heads, which was consistent with the finding from Nekemte Town [25]. This is possibly related to an opportunity of pooling resources from different sources for household consumption among the married households. These findings imply that widowed and uneducated pensioners and pensioners with large family sizes should be given special attention when planning social protection schemes for pension users.

When food supplies are insufficient, household members use coping strategies that compel them to reduce the quality and quantity of foods. This study showed that pension user households in Jimma Town use different coping strategies ranging from selling any household assets to reduction of dietary intake and consumption of low quality foods which is similar with the findings of the study done in Addis Ababa and West Gojjam [18, 19]. Poor dietary intakes in the face of food shortages come from behavioral adaption to overcome the shortage. Coping strategies could undermine the nutrient intakes of children and adolescents within these households having a negative repercussion on their growth [24–26].

Findings reported in this study should be interpreted in the light of the following limitations. Food insecurity measured using HFIAS could lead to social desirability bias as it is based on a self-reported response. However, the potential bias may be on both directions. The bias may overestimate the food insecurity level if the respondents want to get more attention and support. It may also underestimate food insecurity level if the respondents do not have self confidence in disclosing their food security problems to others. Moreover, the scale has been validated in Ethiopia and other developing countries making the possibility of such bias minimal [24]. Due to the cross-sectional nature of the study, the current predictors of food insecurity do not necessarily have cause-effect relationship with food insecurity. Hence, careful interpretation of the findings is critical. The findings of this study can have significant implication for national nutrition programs and informing stakeholders to address the issue nutrition among pension users. The fact that more than 83% of pension users were food insecure in Jimma town implies that further attention of both governmental and non-government organizations should be drawn towards pension user populations.

## Conclusions

Household food insecurity among pension users was remarkably high. Household food insecurity was associated with illiterate household heads, widowed household heads, and household heads employed as guards. The odds of food insecurity among households having large family size (≥ 7) was 3.74 times higher when compared to that of households having less than three family members.

Food insecure households used both consumption and asset based coping strategies such as eating less preferred, lower quality or less expensive foods and receiving donation from relatives or friends.

Therefore, government policies should consider revising the current social protection scheme for pensioners. Special attention should be given to widow pensioners and pensioners with low educational status and with large family sizes.

## Abbreviations

AOR: Adjusted odds ratio
CSA: Central Statistical authority
EDHS: Ethiopian Demographic and Health Survey
EHNRI: Ethiopian Health, Nutrition and Research Institute
HFIAS: Household food insecurity access scale
PCA: Principal Component Analyses
UN: United Nations
UNSPF: United Nation’s Social Protection Floor
